# Metabolic Regulation of Ferroptosis in Breast Cancer

**DOI:** 10.3390/ijms26199686

**Published:** 2025-10-04

**Authors:** Natalija Glibetic, Michael Weichhaus

**Affiliations:** 1 The IDeA Networks of Biomedical Research Excellence (INBRE) Program, School of Natural Sciences and Mathematics, Chaminade University, Honolulu, HI 96816, USA; natalija.glibetic@chaminade.edu; 2 Department of Cell and Molecular Biology, John A Burns School of Medicine, University of Hawaii, 685 Ilalo St, Honolulu, HI 96813, USA; 3 Laboratory of Molecular Cancer Research, School of Natural Sciences and Mathematics, Chaminade University of Honolulu, Honolulu, HI 96816, USA

**Keywords:** ferroptosis, breast cancer, lipid peroxidation, iron metabolism, glutathione metabolism

## Abstract

Breast cancer, a leading global malignancy, exhibits extensive metabolic reprogramming that drives tumorigenesis, therapy resistance, and survival. Ferroptosis, an iron-dependent regulated cell death mechanism characterized by lipid peroxidation, emerges as a promising therapeutic vulnerability, particularly in aggressive subtypes like triple-negative breast cancer (TNBC). This literature review comprehensively explores the metabolic regulation of ferroptosis in breast cancer cells, focusing on how dysregulated pathways modulate sensitivity or resistance. The review will discuss iron homeostasis, including upregulated transferrin receptor 1 (TFR1), diminished ferroportin, mitochondrial dynamics, and ferritinophagy, which catalyze ROS via Fenton reactions. It will examine glutathione (GSH) metabolism through the GPX4-GSH axis, with subtype-specific reliance on cystine import via xCT or de novo cysteine synthesis. Lipid metabolism will be analyzed as the core battleground, highlighting polyunsaturated fatty acid (PUFA) incorporation by ACSL4 promoting peroxidation, contrasted with monounsaturated fatty acid (MUFA) protection via SCD1, alongside subtype adaptations. Further, the review will address tumor microenvironment influences, such as cysteine supply from cancer-associated fibroblasts and oleic acid from adipocytes. Oncogenic signaling (e.g., RAS, mTOR) and tumor suppressors (e.g., p53) will be evaluated for their roles in resistance or sensitivity. Intersections with glucose metabolism (Warburg effect) and selenium-dependent antioxidants will be explored. Therapeutically, the review will consider targeting these nodes with GPX4 inhibitors or iron overload, synergized with immunotherapy for immunogenic cell death. Future directions will emphasize multi-omics integration and patient-derived organoids to uncover subtype-specific strategies for precision medicine in breast cancer.

## 1. Introduction

Breast cancer remains a leading cause of cancer-related mortality worldwide, with rising incidence rates despite therapeutic advances [1]. Ferroptosis, an iron-dependent form of regulated cell death characterized by lipid peroxidation, was first described by Stockwell and colleagues in 2012 as a distinct mechanism from apoptosis, necrosis, or autophagy [2]. This cell death pathway has emerged as a promising therapeutic vulnerability in breast cancer, particularly in aggressive subtypes like triple-negative breast cancer (TNBC) that frequently develop resistance to conventional therapies.

While the core mechanisms of ferroptosis—iron accumulation, lipid peroxidation, and antioxidant system failure—are established, critical knowledge gaps remain regarding how breast cancer cells metabolically reprogram to evade ferroptotic death. Specifically, the field lacks comprehensive understanding of (1) how distinct breast cancer subtypes differentially regulate ferroptosis through metabolic adaptations, (2) the role of the tumor microenvironment in modulating ferroptosis sensitivity through metabolic crosstalk, (3) how oncogenic signaling pathways intersect with ferroptosis regulation to confer therapeutic resistance, and (4) which metabolic nodes represent actionable therapeutic targets for ferroptosis induction.

**Figure 1:**
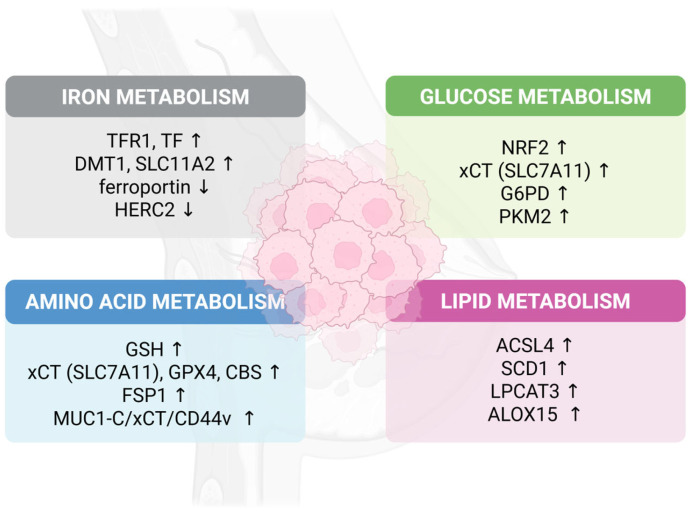
Schematic overview of metabolic pathways regulating ferroptosis in breast cancer. The diagram illustrates key alterations in iron metabolism (**top left**), glucose metabolism (**top right**), amino acid metabolism (**bottom left**), and lipid metabolism (**bottom right**) that influence ferroptosis susceptibility. Upregulated (↑) and downregulated (↓) genes/proteins are indicated, contributing to increased iron accumulation, enhanced antioxidant defenses, altered glucose utilization, and promoted lipid peroxidation, collectively modulating cell death resistance in breast cancer. Abbreviations: ACSL4, acyl-CoA synthetase long-chain family member 4; ALOX15, arachidonate 15-lipoxygenase; CBS, cystathionine beta-synthase; CD44v, CD44 variant; DMT1, divalent metal transporter 1; FSP1, ferroptosis suppressor protein 1; G6PD, glucose-6-phosphate dehydrogenase; GPX4, glutathione peroxidase 4; GSH, glutathione; HERC2, HECT and RLD domain containing E3 ubiquitin protein ligase 2; LPCAT3, lysophosphatidylcholine acyltransferase 3; MUC1-C, mucin 1 C-terminal subunit; NRF2, nuclear factor erythroid 2-related factor 2; PKM2, pyruvate kinase M2; SCD1, stearoyl-CoA desaturase 1; SLC11A2, solute carrier family 11 member 2; SLC7A11, solute carrier family 7 member 11; TF, transferrin; TFR1, transferrin receptor 1; xCT, cystine/glutamate antiporter.

Recent evidence suggests that breast cancer cells exploit multiple metabolic pathways to maintain ferroptosis resistance, including altered iron homeostasis, enhanced glutathione synthesis, lipid metabolism reprogramming, and metabolic support from the tumor microenvironment (Figure 1). However, these adaptations vary significantly across breast cancer subtypes and remain incompletely characterized. For instance, while TNBC shows high baseline ferroptosis sensitivity, certain TNBC subtypes paradoxically upregulate ferroptosis-promoting factors like ACSL4, suggesting complex regulatory mechanisms that balance survival advantages against vulnerability to oxidative death.

This review addresses these knowledge gaps by providing a comprehensive analysis of the metabolic networks governing ferroptosis in breast cancer. We systematically examine iron metabolism, glutathione and amino acid regulation, lipid metabolism alterations, and the unique metabolic reprogramming of breast cancer subtypes. Furthermore, we explore how the breast tumor microenvironment creates ferroptosis-resistant niches through metabolic crosstalk and evaluate emerging therapeutic strategies that exploit these metabolic vulnerabilities. By integrating recent discoveries in ferroptosis biology with breast cancer metabolism, this review aims to identify actionable metabolic targets and inform precision medicine approaches for ferroptosis-based therapies.

## 2. Iron Metabolism: The Catalytic Foundation

### 2.1. Iron Uptake and Regulation

Iron metabolism drives ferroptosis through the labile iron pool (LIP), which catalyzes Fenton reactions generating hydroxyl radicals. Transferrin receptor 1 (TFR1), frequently overexpressed in breast cancer, shows a clear progression from benign to invasive carcinomas [3], with particularly high expression in high-grade and estrogen receptor (ER)-negative tumors [3,4]. This overexpression correlates with poor outcomes and tamoxifen resistance in ER+ patients [4], though the mechanistic link between TFR1 levels and therapy resistance remains incompletely understood.

Beyond TFR1, breast cancer cells employ multiple iron acquisition strategies. Divalent metal transporter 1 (DMT1) mediates non-transferrin-bound iron uptake, particularly in TNBC [5], while loss of ferroportin—the only iron exporter—creates intracellular accumulation [6]. The tumor microenvironment further modulates iron availability through interleukin-6 (IL-6) paracrine signaling, contributing to chemoresistance [7], though whether this is cause or consequence of treatment failure requires clarification. Alterations in endosomal pH can impair iron release from transferrin, disrupting homeostasis and favoring progression [8].

While TFR1 upregulation is consistently observed across breast cancer subtypes (strong evidence from >20 studies), the functional significance remains subject to debate. The paradox that cancer cells accumulate iron despite its pro-ferroptotic effects suggests that either (1) iron provides selective advantages outweighing ferroptosis risk, (2) concurrent antioxidant upregulation neutralizes iron toxicity, or (3) iron accumulation is a passenger alteration. The failure of iron chelators as monotherapy [6] supports the second hypothesis, indicating that targeting iron alone is insufficient without disrupting compensatory mechanisms.

### 2.2. Mitochondrial Iron Dynamics

Mitochondria play a crucial role in ferroptosis regulation as the primary site of iron utilization and ROS generation. Cancer cells accumulate iron in mitochondria, increasing their susceptibility to oxidative stress and ferroptotic death [9,10]. In TNBC specifically, dysregulated mitochondrial iron transport enhances ferroptosis vulnerability under metabolic stress [11]. CDGSH iron sulfur domain 1 (CISD1) emerges as a key protective factor—its knockdown significantly increases mitochondrial ferrous iron and promotes ferroptotic death [12].

While mitochondrial iron accumulation is documented across breast cancer types, the directionality remains unclear. Does increased mitochondrial iron drive transformation or result from it? The tissue-specific role of CISD1 is particularly puzzling, as its protective function varies dramatically between cell lines [12]. This variability suggests that mitochondrial iron’s contribution to ferroptosis may be context-dependent rather than universally applicable, limiting therapeutic targeting strategies.

### 2.3. Iron Storage and Release

The balance between iron storage and release critically determines ferroptosis susceptibility. Nuclear receptor coactivator 4 (NCOA4)-mediated ferritinophagy releases iron from ferritin storage, increasing the LIP and sensitizing cells to ferroptosis [13]. HERC2 downregulation enhances this process by stabilizing NCOA4 [14]. Breast cancer cells exploit this system—while maintaining iron storage for survival, they become vulnerable to forced ferritinophagy. Sorafenib-loaded nanoparticles effectively trigger this vulnerability, upregulating NCOA4 to induce ferroptosis while boosting immune responses [15].

Recent evidence shifts focus to lysosomal iron stores as the primary ferroptosis initiator, with lipid peroxidation beginning in lysosomes before propagating to other organelles [16]. This compartmentalized view of iron-induced death challenges the traditional cytoplasmic-centric model of ferroptosis.

The ferritinophagy pathway presents a therapeutic paradox in that cancer cells require NCOA4-mediated iron release for proliferation, yet this same pathway sensitizes them to ferroptosis. The recent lysosomal iron discovery [16] fundamentally challenges this understanding, suggesting that targeting lysosomal rather than cytoplasmic iron may be more effective. However, the clinical translation remains limited by our inability to selectively induce ferritinophagy in cancer versus normal cells, explaining why ferritinophagy-targeting agents remain in preclinical stages.

## 3. Glutathione Metabolism and Amino Acid Regulation

### 3.1. The Central GPX4-GSH Axis

Glutathione (GSH) serves as the cornerstone of ferroptosis regulation through its role as a cofactor for glutathione peroxidase 4 (GPX4), which converts lipid peroxides into non-toxic lipid alcohols [17,18]. When GSH is depleted, GPX4 becomes inactivated, leading to lethal lipid peroxide accumulation [19].

The xCT/GPX4 axis becomes upregulated in TNBC, with cells developing an “addiction” to this cystine import-glutathione defense system [20]. TNBC cells use the MUC1-C/xCT/CD44v complex to maintain glutathione levels and resist ferroptosis [21]. Mitochondria-targeted strategies show that iron overload combined with GSH depletion disrupts redox homeostasis and promotes ferroptosis in TNBC cells [11].

The GPX4-GSH axis represents the best-validated ferroptosis mechanism, with consistent evidence across >50 breast cancer studies. However, the clinical translation faces two major obstacles: (1) GPX4 inhibitors like RSL3 show poor bioavailability (t½ < 2 h) and hepatotoxicity at therapeutic doses, and (2) the “addiction” to xCT/GPX4 in TNBC paradoxically coexists with high baseline ferroptosis sensitivity. This suggests compensatory mechanisms we don’t fully understand, potentially explaining why GPX4-targeting monotherapies have yet to enter clinical trials for breast cancer.

### 3.2. Cysteine Transport and Synthesis

Cysteine availability rate-limits GSH synthesis through two distinct pathways. The system xc^−^ transporter (SLC7A11/xCT) imports cystine, which is reduced to cysteine for GSH synthesis [22]. In breast cancer, xCT upregulation reflects dependency on extracellular cysteine [23]. Inhibition with erastin or imidazole ketone erastin (IKE) depletes GSH and induces ferroptosis [22,24]. Tumor-associated macrophages (TAMs) in TNBC secrete TGFβ1, enhancing gamma-glutamyltransferase 1 (GGT1) transcription to increase cysteine availability and suppress ferroptosis [25]. Luminal A cancers specifically depend on xCT to protect against CDK4/6 inhibitor-induced ferroptosis [26].

Alternatively, the transsulfuration pathway synthesizes cysteine de novo through cystathionine beta-synthase (CBS) and cystathionine gamma-lyase (CTH) [27]. This pathway becomes critical when extracellular cysteine is limited [28]. Basal-like breast cancers exhibit high CBS activity, creating “cysteine addiction” that confers ferroptosis resistance—silencing CBS sensitizes these cells to death [29]. Low cytokine-like 1 (CYTL1) levels suppress CBS, increasing ferroptosis risk when cystine import is blocked [30].

Beyond import and synthesis, lysosomal cysteine storage provides a third ferroptosis resistance mechanism. Lysosomal cystine shortage specifically triggers ferroptosis through ATF4-mediated stress responses [31]. Breast cancer cells store cysteine via the MFSD12 transporter and release it through cystinosin (CTNS) to maintain GSH levels [32]. Enhancing lysosomal cystine with synthetic mRNA (CysRx) paradoxically increases ferroptosis and suppresses tumor growth in vivo [31], suggesting that disrupting lysosomal cysteine homeostasis represents a therapeutic vulnerability.

A clear subtype-specific dichotomy emerges: TNBC and drug-resistant subtypes primarily rely on CBS-driven transsulfuration, while luminal A and HER2+ subtypes depend on xCT-mediated import. This differential dependency provides precision targeting opportunities, yet clinical exploitation remains elusive. The paradox that both pathways can be simultaneously upregulated in some cancers suggests redundancy that may limit single-pathway targeting. Moreover, the TAM-mediated cysteine supply [22] indicates that tumor microenvironment contributions may override cancer cell-autonomous vulnerabilities. The lysosomal cysteine compartment adds yet another layer of redundancy [31,32], potentially explaining the limited success of targeting cytoplasmic cysteine pathways alone.

### 3.3. Alternative Cysteine Actions

Cysteine and homocysteine can act as alternative thiol donors for GPX4, providing GSH-independent ferroptosis protection [33]. Additionally, cysteine may inhibit ferroptosis through the ferroptosis suppressor protein 1 (FSP1) pathway [34]. FSP1 converts coenzyme Q10 (CoQ10) to ubiquinol using NADPH, creating a parallel antioxidant system at the plasma membrane [35,36].

Cysteine contributes to this FSP1-CoQ10 system through the pantothenate-coenzyme A synthesis pathway. Labeled cystine incorporates into both glutathione and coenzyme A synthesis [37], suggesting cysteine’s dual protective role against ferroptosis [34].

The discovery of GSH-independent ferroptosis protection mechanisms fundamentally challenges the GPX4-centric view of ferroptosis regulation. The FSP1-CoQ10 system’s existence [35,36] explains why GPX4 inhibition alone often fails to induce complete ferroptosis. However, the relative contribution of these alternative pathways in breast cancer remains unclear. The pantothenate connection [37], is particularly intriguing but lacks breast cancer-specific validation, representing a knowledge gap that could explain therapeutic resistance patterns.

### 3.4. Nucleotide Metabolism Connections

Nucleotide biosynthesis competes with ferroptosis regulation for GSH resources. Ribonucleotide reductase (RNR) consumes significant GSH for deoxyribonucleotide production, reducing GPX4-available GSH and heightening ferroptosis vulnerability [38]. The p53-p21 axis modulates this competition by suppressing RNR under stress [39]. Nucleotide supplementation can restore proliferation in cysteine-deprived breast cancer cells, confirming direct competition between these pathways [40]. mTORC1 further coordinates this balance by linking GPX4 synthesis to cysteine availability [41].

The GSH competition between nucleotide synthesis and ferroptosis defense represents an underexploited therapeutic vulnerability. While the mechanism is well-established [38,39,40,41], clinical translation remains absent. The key challenge lies in selectively disrupting this balance in cancer versus normal cells, as both require nucleotide synthesis. The finding that nucleotide supplementation rescues cysteine-deprived cells [40] suggests combination therapies blocking both cysteine import and nucleotide synthesis might overcome resistance, though toxicity concerns have prevented clinical advancement (Table 1).

## 4. Lipid Metabolism: The Primary Battleground

### 4.1. Polyunsaturated Fatty Acid (PUFA) Synthesis and Incorporation

Polyunsaturated fatty acids (PUFAs), particularly arachidonic acid (AA) and adrenic acid (AdA), serve as primary ferroptosis substrates when incorporated into membrane phospholipids. In breast cancer, PUFA metabolism shows marked dysregulation. Research reveals significant PUFA reduction in malignant phosphoglycerides, attributed to impaired fatty acid desaturases (FADS1/2) [47]. Estrogen further disrupts this balance by upregulating ELOVL2 through ERα, enhancing AA elongation to AdA and shifting membrane omega-6 profiles [48]. These alterations correlate with cancer progression and diminished survival [49].

Dietary omega-3 PUFAs offer potential protection by modulating membrane composition and countering omega-6 dominance [50,51]. However, the clinical translation of dietary interventions remains inconclusive.

The PUFA synthesis disruption in breast cancer presents a fundamental paradox—reduced PUFA content should theoretically protect against ferroptosis, yet these cells remain vulnerable. This suggests that either (1) the specific PUFA species matter more than total content, (2) subcellular PUFA distribution rather than total levels determines sensitivity, or (3) compensatory mechanisms override PUFA depletion effects. The estrogen-ELOVL2-AdA axis [48] provides a mechanistic link between hormonal signaling and ferroptosis susceptibility, potentially explaining ER+ tumors’ lower baseline sensitivity. However, targeting PUFA synthesis directly has shown limited success, likely due to dietary PUFA compensation.

### 4.2. ACSL4: The Critical Enzyme

Acyl-CoA synthetase long-chain family member 4 (ACSL4) emerges as the master regulator of ferroptosis sensitivity by preferentially activating AA and AdA for incorporation into phospholipids [52,53]. ACSL4’s role in breast cancer is paradoxically subtype-dependent: it inhibits progression in ER+ tumors while promoting proliferation and invasion in ER-negative cancers [54,55]. ACSL4 expression strongly correlates with ferroptosis sensitivity, positioning it as a predictive biomarker [52,56]. ACSL4 deficiency confers ferroptosis resistance, though targeted lipid delivery can bypass this protection [57].

ACSL4 presents the most striking paradox in breast cancer ferroptosis; it is preferentially expressed in basal-like breast cancer [56], predicting higher ferroptosis sensitivity, yet these tumors survive and proliferate aggressively. This suggests that ACSL4 provides selective advantages that outweigh ferroptosis vulnerability, possibly through enhanced membrane remodeling or signaling lipid production. The dual tumor-suppressive (ER+) versus oncogenic (ER−) roles [54,55] indicate context-dependent functions we don’t fully understand. While ACSL4 is the most validated ferroptosis biomarker, its therapeutic targeting remains challenging due to this functional duality and the lack of selective inhibitors that spare normal tissue function.

### 4.3. Lipid Peroxidation Mechanisms

Enzymatic lipid peroxidation involves arachidonate 15-lipoxygenase (ALOX15), which oxygenates AA-containing phospholipids at the 15th carbon, producing lipid hydroperoxides that disrupt membrane integrity [58]. In breast cancer, ALOX15 expression predicts ferroptosis sensitivity and is included in prognostic models [59,60]. TNBC cells exhibit unique iron and glutathione profiles that amplify both enzymatic and non-enzymatic peroxidation [61]. In addition to lipoxygenases, heme oxygenases contribute to lipid peroxidation in breast cancer cells [62].

While ALOX15 is consistently identified as a ferroptosis mediator, its expression varies dramatically across breast cancer subtypes without clear correlation to clinical outcomes [59,60]. The relative contribution of enzymatic versus non-enzymatic peroxidation remains undefined. Iron-catalyzed Fenton reactions may dominate in high-iron environments like TNBC, while enzymatic pathways prevail in iron-normal contexts. The context-dependent nature of ALOX15 function suggests it may serve as a prognostic marker rather than a therapeutic target. The lack of selective ALOX15 inhibitors that spare normal inflammatory resolution further limits clinical translation.

### 4.4. Monounsaturated vs. Polyunsaturated Fatty Acids

The SREBP1-SCD axis drives monounsaturated fatty acid (MUFA) production, converting saturated fatty acids to oleic acid, which protects against ferroptosis [63,64]. SCD1 upregulation correlates with worse prognosis in breast cancer, while its inhibition sensitizes cells to ferroptosis [64]. Conversely, ACSL3 activates MUFAs for incorporation via membrane-bound O-acyltransferases (MBOAT1/2), which transfer MUFA-CoAs into phospholipids, reducing peroxidizable substrates [65,66]. MBOAT1, regulated by ER signaling, is highly expressed in breast cancer [66].

Despite ACSL4’s high expression in basal-like breast cancer predicting ferroptosis sensitivity [67], these cells paradoxically survive. Similarly, a TNBC subset shows elevated FADS1/2 expression, rendering them susceptible to ferroptosis inducers [68].

The MUFA/PUFA balance represents a druggable metabolic switch, yet clinical exploitation remains elusive. The paradoxical survival of ACSL4-high/PUFA-rich TNBC cells [67] suggests unknown compensatory mechanisms, possibly through enhanced MUFA production or alternative antioxidant systems. The ER-MBOAT1 connection [66] provides rationale for combining ER antagonists with ferroptosis inducers in hormone-positive disease. However, the fundamental question remains: why do cancer cells maintain high ACSL4/PUFA levels despite ferroptosis risk? This likely reflects PUFAs’ essential roles in signaling and membrane dynamics that outweigh death susceptibility. SCD1 inhibitors show promise but face challenges with systemic toxicity due to MUFA requirements in normal tissues.

### 4.5. Lipid Availability and Trafficking

Extracellular lipid limitation paradoxically increases ferroptosis sensitivity despite reducing total PUFA levels [69]. Under lipid starvation, adipose triglyceride lipase (ATGL) mobilizes stored triglycerides, liberating PUFAs that are directly incorporated into membrane phospholipids rather than being stored in protective lipid droplets [70,71,72]. This creates highly peroxidizable phosphatidylethanolamine-AA/AdA species that serve as primary ferroptosis substrates.

The LPCAT enzyme family determines membrane PUFA incorporation: LPCAT3 enhances ferroptosis by selectively incorporating AA into phospholipids [73], while LPCAT1 and LPCAT4/MBOAT2 confer resistance through saturated fatty acid and MUFA incorporation [74,75]. The PLA2 family provides the hydrolytic component, with iPLA2β limiting ferroptosis by cleaving oxidized chains [76]. ACSL1 facilitates protective lipid droplet formation, sequestering PUFAs from vulnerable membranes [77,78].

The lipid availability paradox—where nutrient limitation increases death susceptibility—fundamentally challenges cancer biology dogma. This mechanism [69] explains why metabolically stressed tumor regions show enhanced ferroptosis sensitivity, offering therapeutic opportunities through dietary interventions or lipid uptake inhibition. However, the complexity of lipid trafficking networks (>20 enzymes with overlapping functions) makes targeted intervention challenging. The protective role of lipid droplets [77,78] suggests that combination strategies disrupting both storage and membrane incorporation might overcome resistance. Yet clinical translation remains limited by our inability to predict which tumors rely on stored versus dietary lipids, and normal tissues’ requirement for these same pathways.

## 5. Breast Cancer-Specific Metabolic Reprogramming

### 5.1. Breast Tumor Microenvironment Factors

*Cancer-Associated Fibroblasts (CAFs)*. CAFs supply cysteine and glutathione while secreting IL-6 to enhance hepcidin expression and increase iron availability [79,80]. They produce cysteine de novo via CBS, directly supporting cancer cell GSH synthesis [81]. CAF-derived lactate induces histone lactylation, enhancing ZFP64 expression and promoting ferroptosis resistance in TNBC, contributing to doxorubicin resistance [82].

*Cancer-Associated Adipocytes*. Mammary adipocytes protect TNBC cells by secreting oleic acid, which is converted via ACSL3 into membrane phospholipids, displacing PUFAs and reducing peroxidation substrates [83,84]. Co-cultured breast cancer cells show elevated MUFA/PUFA ratios, creating oxidation-resistant membranes [83,85]. High-fat diet exposure downregulates ACSL4 expression, promoting ferroptosis resistance [86].

*Immune Components*. Tumor-associated macrophages (TAMs) show differential ferroptosis susceptibility—M1 macrophages resist through iNOS/nitric oxide, while M2 macrophages remain sensitive [87,88]. CD8+ T cells release IFNγ and arachidonic acid, sensitizing tumors to ferroptosis by inhibiting xCT and promoting ACSL4-mediated PUFA incorporation [89,90]. However, CAF-derived factors can override these pro-ferroptotic signals.

*Matrix and Integration*. α6β4 integrin protects against ferroptosis during matrix detachment by maintaining GPX4 expression through Src/STAT3 signaling [91,92].

The microenvironment creates a ferroptosis-resistant sanctuary that likely explains clinical treatment failures. The CAF-adipocyte metabolic support network provides redundant protection mechanisms—even if cancer cell-autonomous pathways are targeted, stromal supply of cysteine, glutathione, and protective lipids maintains resistance. The paradox that immune cells can both promote (CD8+ T cells) and protect against (M2 macrophages) ferroptosis [87,88,89,90] suggests that immunotherapy outcomes may depend on the ferroptosis status of both tumor and immune compartments. Most critically, no current therapies can selectively disrupt stromal–tumor metabolic coupling without affecting normal tissue homeostasis, representing a major barrier to clinical translation.

### 5.2. Breast Cancer Subtype Variations

TNBC exhibits the highest ferroptosis sensitivity, particularly the luminal androgen receptor (LAR) subtype, due to upregulated oxidized phosphatidylethanolamines (OxPE) and exhausted PUFA pools [93,94]. The LAR subtype’s hypersensitivity to GPX4 inhibitors correlates with distinct lipid profiles and glutathione metabolism alterations [95]. However, TNBC heterogeneity creates variable responses—mesenchymal subtypes show moderate sensitivity with enriched iron metabolism, while immunomodulatory subtypes exhibit minimal ferroptosis features (Table 2) [96].

ER-positive breast cancers (Luminal A/B) demonstrate low baseline ferroptosis sensitivity (low FERscores) [97]. Estrogen signaling upregulates MBOAT1, suppressing ferroptosis, but ER antagonists reverse this protection by downregulating MBOAT1 [98]. A ferroptosis-associated gene signature predicts treatment response across risk groups [99,100].

HER2-enriched tumors show context-dependent sensitivity—moderate baseline vulnerability increases substantially in trastuzumab-resistant states. Lapatinib-tolerant persister cells exhibit GPX4 dependency, creating opportunities for combination therapy [101,102].

The subtype-specific ferroptosis patterns reveal a fundamental inconsistency—TNBC’s high sensitivity should confer growth disadvantage, yet these tumors are the most aggressive. This paradox suggests that either (1) ferroptosis-sensitive cells are eliminated during tumor evolution, leaving resistant clones; (2) the microenvironment provides sufficient protection to override intrinsic sensitivity; or (3) we’re measuring sensitivity incorrectly in vitro. The LAR subtype’s extreme sensitivity [93,94,95] offers the clearest therapeutic window, yet no LAR-specific ferroptosis trials exist. The finding that therapy resistance (tamoxifen, trastuzumab) increases ferroptosis vulnerability [101,102] provides immediate translational opportunities for combination strategies in relapsed disease.

### 5.3. Oncogene and Tumor Suppressor Influences

*RAS Pathway*. RAS activation creates multilayered ferroptosis resistance. The RAS-RAF-MEK-ERK cascade upregulates xCT through ETS1 phosphorylation [103,104], while activating NRF2-dependent antioxidant programs [105,106]. The FASN-HIF1α axis provides additional protection—FASN binds HIF1α, enhancing xCT transcription while promoting MUFA synthesis [107,108]. FSP1 regulation through NRF2 establishes GPX4-independent protection via CoQ10 reduction [109,110].

*mTOR Pathway*. mTORC1 inhibits ferroptosis through the SREBP1-SCD1 axis, promoting MUFA synthesis that reduces membrane peroxidation [111,112]. Additionally, mTOR suppresses ferritinophagy by inhibiting NCOA4, limiting free iron [111,112,113], while activating the p62-KEAP1-NRF2 axis through p62 phosphorylation [114,115]. Paradoxically, mTOR inhibition can protect against ferroptosis by activating glutaminolysis, maintaining NADPH for antioxidant defense [116].

*p53*. p53 promotes ferroptosis through multiple mechanisms: direct xCT transcriptional repression [117], SAT1 upregulation leading to ALOX15-mediated lipid peroxidation [118], and GLS2 induction depleting glutamine pools [119]. The S47 p53 variant found in African populations shows impaired GLS2 transactivation, conferring ferroptosis resistance [120].

*Other Suppressors*. BRCA1 deficiency creates differential responses—erastin resistance but GPX4 inhibitor sensitivity [121]. PTEN loss activates AKT-GSK3β-NRF2-xCT, promoting resistance [122,123]. RB deficiency paradoxically increases sensitivity through E2F-mediated ACSL4 upregulation [124].

The oncogene-suppressor ferroptosis network reveals therapeutic complexities (Table 3). RAS pathway targeting faces redundancy—blocking one resistance mechanism (e.g., xCT) is compensated by others (FSP1, FASN). The mTOR paradox, where both activation and inhibition can prevent ferroptosis [112,116], suggests context-dependent effects we don’t fully understand. Most significantly, p53’s ferroptosis-promoting role [117,118,119] contradicts its loss in aggressive cancers. This likely reflects the dominance of oncogenic resistance pathways over tumor suppressor sensitization. The BRCA1 differential response [121] offers immediate clinical relevance for PARP inhibitor combinations, yet remains unexploited. These findings suggest single-pathway targeting will fail; successful strategies must simultaneously disrupt multiple resistance mechanisms.

## 6. Additional Metabolic Pathways

### 6.1. Glucose Metabolism Connections

**Figure 2:**
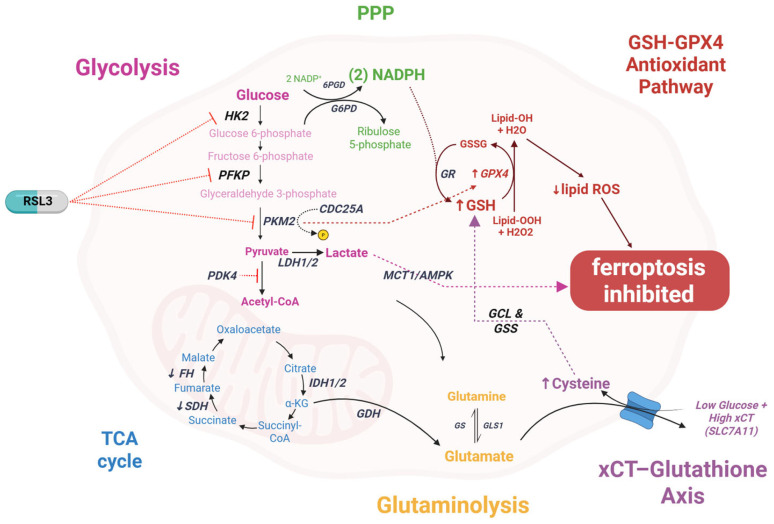
Schematic representation of ferroptosis inhibitor RSL3 actions on glycose metabolism pathways modulating ferroptosis sensitivity in breast cancer cells. The diagram highlights glycolysis (pink), TCA cycle (blue), pentose phosphate pathway (PPP, purple), glutaminolysis (orange), and the GSH-GPX4 antioxidant pathway (red), illustrating their interplay in ferroptosis regulation. Key enzymes (e.g., HK2, PFK, PKM2, LDH1/2, IDH1/2, G6PD, GR, GPX4) and metabolites (e.g., NADPH, GSH, GSSG, lipid ROS) are shown, with arrows indicating activation or inhibition. RSL3 inhibits ferroptosis by targeting the xCT-Glutathione Axis (purple), reducing cysteine uptake and glutathione synthesis under low glucose and high xCT (SLC7A11) conditions. Abbreviations: α-KG, alpha-ketoglutarate; CDC25A, cell division cycle 25A; GDH, glutamate dehydrogenase; GLS1, glutaminase 1; MCT1, monocarboxylate transporter 1; PDK4, pyruvate dehydrogenase kinase 4.

Glucose metabolism critically modulates ferroptosis through NADPH generation and redox balance (Figure 2). The pentose phosphate pathway (PPP), via glucose-6-phosphate dehydrogenase (G6PD), produces NADPH essential for GSH regeneration and GPX4 function [138,139]. Paradoxically, NADPH also fuels NADPH oxidases that generate ROS, creating opposing ferroptosis effects [140]. In breast cancer, high xCT expression coupled with glucose deprivation triggers both ferroptosis and disulfidptosis—a novel death mechanism involving aberrant disulfide crosslinking when NADPH is depleted [141,142].

Glycolytic alterations affect ferroptosis sensitivity: PDK4 blocks pyruvate entry into the TCA cycle, conferring resistance [143], while ferroptosis inducers downregulate key glycolytic enzymes (HK2, PFKP, PKM2), increasing tumor vulnerability [144]. Our own research demonstrated that glucose deprivation in MCF-7 and T47D cells activates ferroptosis through NRF2 signaling [145]. The lactate/MCT1/AMPK axis shows context-dependent effects, promoting ferroptosis in normal environments but conferring resistance in acidic tumor microenvironments [146].

The glucose–ferroptosis relationship presents a therapeutic dilemma. While glucose restriction sensitizes cells to ferroptosis [141,145], tumors adapt through enhanced PPP flux or alternative NADPH sources (folate metabolism, IDH mutations). The discovery of disulfidptosis [141,142] reveals that targeting glucose metabolism can trigger multiple death pathways simultaneously, potentially overcoming resistance. However, the dual role of NADPH—protecting via GSH while promoting ROS via NOX—makes prediction of net effects challenging. The lactate paradox [146], where the same metabolite has opposite effects depending on pH, exemplifies why metabolic interventions fail clinically. GLUT inhibitors remain in preclinical development due to inability to selectively target cancer metabolism without affecting normal glucose homeostasis.

### 6.2. Selenium and Antioxidant Systems

Beyond glutathione, breast cancer cells employ multiple antioxidant systems. Selenium’s incorporation into GPX4 is essential for its function, with sodium selenite paradoxically inducing ferroptosis in TNBC through ATM kinase activation despite being an antioxidant cofactor [147,148]. The thioredoxin reductase system provides GSH-independent protection—its inhibition enhances radiation sensitivity in breast cancer stem cells [64].

The FSP1-CoQ10-NAD(P)H pathway operates independently of GPX4, reducing ubiquinone to ubiquinol at the plasma membrane [35,149]. However, the UBIAD1/CoQ10 system presents a striking paradox: UBIAD1 expression and CoQ10 levels, despite their antioxidant function, actually enhance ferroptosis sensitivity in breast cancer [150,151]. High UBIAD1/CoQ10 cells show decreased GPX4 synthesis and reduced FSP1 levels, with UBIAD1-low patients exhibiting significantly shorter survival, particularly in TNBC [150]. This counterintuitive finding suggests that elevated CoQ10 may disrupt the balance between different antioxidant systems, potentially through negative feedback on GPX4/FSP1 expression or by altering cellular redox setpoints that make cells more vulnerable to acute oxidative challenges.

Additional protective mechanisms include the GCH1–tetrahydrobiopterin (BH4) axis, which prevents ferroptosis through lipid remodeling [152], and vitamin E derivatives like D-α-tocopherol succinate that paradoxically induce rather than prevent ferroptosis in drug-resistant cells [153].

The non-glutathione antioxidant systems reveal unexpected complexities that challenge therapeutic targeting. The UBIAD1/CoQ10 paradox [150,151]—where antioxidant elevation increases death susceptibility—suggests that cellular redox homeostasis operates within narrow boundaries, and both deficiency and excess can trigger ferroptosis. This may explain why antioxidant supplementation trials often fail or worsen cancer outcomes. The selenium paradox [147,148], where the same element can prevent or promote ferroptosis depending on dose and form, further complicates therapeutic development. The existence of multiple parallel systems (FSP1, thioredoxin, BH4) explains why GPX4 inhibition alone rarely achieves complete ferroptosis. Clinical translation requires either simultaneous inhibition of all systems (likely toxic) or exploitation of paradoxical responses like UBIAD1, though mechanisms remain incompletely understood.

## 7. Future Directions and Challenges

### 7.1. Multi-Omics Integration and Tumor Organoids

Multi-omics integration has revealed subtype-specific ferroptosis vulnerabilities in breast cancer. A lipid metabolism–ferroptosis signature successfully stratified HR+ patients into risk groups with distinct therapeutic responses [154]. Quantification of ferroptosis pathway status uncovered TNBC-specific fatty acid metabolism alterations predictive of treatment outcomes [155].

Patient-derived organoids (PDOs) provide functional validation of multi-omics findings [156]. HER2+ organoids revealed that combined anti-FGFR4/anti-HER2 therapy triggers synergistic ferroptosis [157]. Tamoxifen induces ferroptosis in MCF-7 organoids through lipid metabolism disruption [158], while minimal residual disease organoids exhibit dysregulated lipid metabolism with elevated oxidative stress, identifying a therapeutic window [159].

While multi-omics approaches generate comprehensive datasets, clinical translation remains limited. The identified signatures [154,155] lack prospective validation and standardized thresholds for patient stratification. PDO models [157,158,159], though promising, fail to recapitulate the tumor microenvironment’s metabolic contributions—a critical limitation given our findings that CAFs and adipocytes provide ferroptosis protection (Section 5.1). The correlation between multi-omics signatures and actual ferroptosis occurrence in patients remains unvalidated. Most critically, these approaches identify associations rather than causal relationships, limiting therapeutic targeting. The field requires functional validation studies linking specific metabolic signatures to ferroptosis sensitivity in clinical specimens, not just cell lines or organoids.

### 7.2. Immunotherapy Synergy

**Figure 3:**
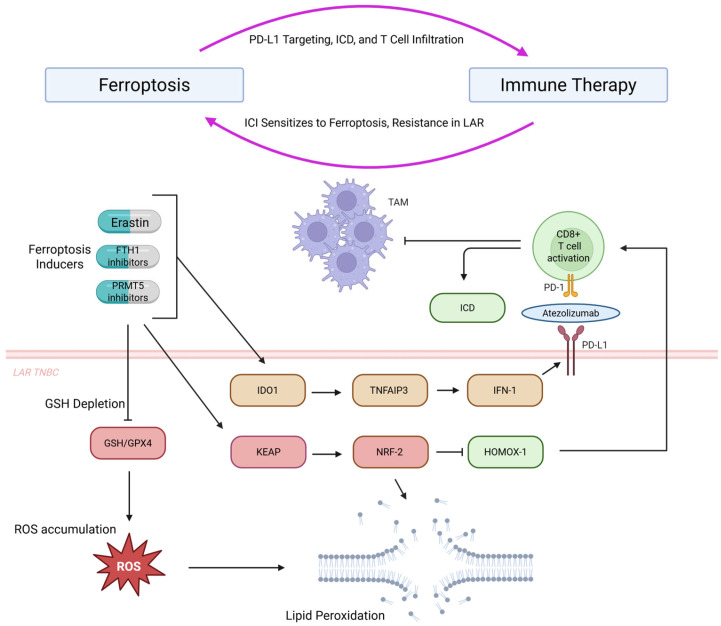
Schematic representation of the interplay between ferroptosis and immunotherapy in luminal androgen receptor (LAR) triple-negative breast cancer (TNBC). The diagram illustrates how ferroptosis inducers (Erastin, FTH1 inhibitors, PRMT5 inhibitors) and GSH depletion trigger ROS accumulation and lipid peroxidation, mediated by GSH/GPX4, KEAP, and NRF2 pathways. Immune checkpoint inhibitors (ICIs) sensitize ferroptosis resistance in LAR TNBC, enhancing tumor-associated macrophage (TAM) activity and immunogenic cell death (ICD) through IDO1, TNFAIP3, IFN1, and HMOX1 signaling. This activates CD8+ T cells and PD-1/PD-L1 targeting (Atezolizumab), promoting immune therapy efficacy via PD-L1 targeting, ICD, and T cell infiltration. Abbreviations: FTH1, ferritin heavy chain 1; GSH, glutathione; GPX4, glutathione peroxidase 4; ICD, immunogenic cell death; IDO1, indoleamine 2,3-dioxygenase 1; KEAP, Kelch-like ECH-associated protein; NRF2, nuclear factor erythroid 2-related factor 2; PRMT5, protein arginine methyltransferase 5; ROS, reactive oxygen species; TAM, tumor-associated macrophage; TNFAIP3, tumor necrosis factor alpha-induced protein 3.

Ferroptosis induction synergizes with immune checkpoint inhibitors (ICIs) through multiple mechanisms (Figure 3). Ferroptotic cancer cells upregulate PD-L1 via IFNγ signaling, creating ideal ICI targets [160]. Cyclophosphamide, a ferroptosis-promoting chemotherapeutic, increases PD-L1 in TNBC immune infiltrates [160]. The LAR TNBC subtype shows high ferroptosis resistance correlating with poor ICI response, while targeting GPX4 with RSL3 sensitizes these cells and enhances T-cell infiltration [161].

PRMT5 emerges as a key resistance mechanism, methylating KEAP1 to stabilize NRF2 and suppress ferroptosis. PRMT5 inhibition combined with anti-PD-1 achieves 60–80% tumor growth inhibition versus monotherapy [162]. Natural compounds like toxicarioside H and statins deplete GSH to trigger ferroptosis in TNBC [163,164]. Targeted delivery strategies using PD-1-coated nanoparticles achieve 90% tumor inhibition with anti-PD-L1 combination [165].

While ferroptosis–immunotherapy synergy shows impressive preclinical results (60–90% tumor inhibition) [162,165], no clinical trials have validated these findings. The major paradox—that ferroptosis induces immunogenic cell death yet simultaneously impairs CD8+ T cells through CD36-mediated lipid uptake—remains unresolved. The PD-L1 upregulation mechanism [160] could theoretically enhance ICI response, but ferroptotic damage to effector T cells might negate benefits. PRMT5 inhibitors are in Phase I trials for other cancers, not specifically for ferroptosis enhancement. The field lacks biomarkers to identify patients who would benefit from combination therapy versus those at risk for immune dysfunction. Most critically, the optimal sequencing—simultaneous versus sequential ferroptosis induction and ICI—remains undefined, with opposing rationales for each approach.

### 7.3. Photodynamic-Ferroptosis Synergy Treatment

**Figure 4:**
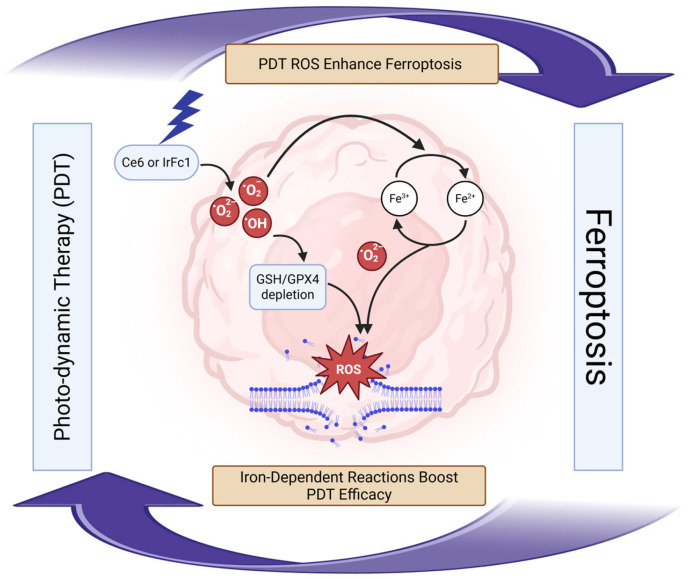
Schematic illustration of the interplay between photodynamic therapy (PDT) and ferroptosis in cancer cells. The diagram depicts how PDT, utilizing Ce6 or IrFc1 photosensitizers (blue), generates reactive oxygen species (ROS) that enhance ferroptosis (purple arrows). Key processes include the conversion of Fe^3+^ to Fe^2+^, production of hydroxyl radicals (•OH), and depletion of GSH/GPX4, leading to lipid peroxidation and ROS accumulation (red). Iron-dependent reactions amplify PDT efficacy, creating a feedback loop that boosts ferroptosis-mediated cell death. Abbreviations: GSH, glutathione; GPX4, glutathione peroxidase 4; ROS, reactive oxygen species.

Photodynamic therapy (PDT) directly triggers ferroptosis through reactive oxygen species generation that overwhelms the GSH/GPX4 system [166] (Figure 4). PDT achieves tumor inhibition rates exceeding 95% when combined with ferroptosis inducers, addressing PDT’s oxygen dependence and ferroptosis’s requirement for specific cellular conditions [167,168].

Chlorin e6 (Ce6) and protoporphyrin IX serve as primary photosensitizers, generating singlet oxygen that depletes GSH and downregulates GPX4 [169,170]. Novel iridium-ferrocene conjugates (IrFc1) provide self-amplifying mechanisms where endogenous ROS enhance photosensitization specifically in TNBC [171]. The combination creates a feedback loop where PDT-generated ROS enhance ferroptosis while iron-dependent reactions improve PDT efficacy [166,172].

Despite impressive preclinical results (>95% tumor inhibition) [167,168], PDT-ferroptosis combination faces insurmountable clinical barriers. PDT’s fundamental limitation—light penetration restricted to <1 cm tissue depth—makes it unsuitable for most breast cancers except superficial recurrences. The oxygen dependence of both PDT and ferroptosis creates a therapeutic paradox in hypoxic tumor cores where treatment is most needed. While 217 publications appeared in 2023 alone [173], none report clinical trials. The photosensitizers showing best ferroptosis synergy (Ce6, IrFc1) lack FDA approval for any indication. Most critically, the simultaneous requirement for light delivery, adequate tissue oxygenation, and ferroptosis-permissive conditions may never align in clinical tumors. This approach likely remains limited to ex vivo treatment of surgical margins or palliative management of cutaneous metastases.

## 8. Conclusions

Ferroptosis represents both a promising therapeutic target and a cautionary tale of biological complexity in breast cancer. Our analysis reveals that breast cancer cells navigate a precarious balance, maintaining sufficient iron and PUFAs for proliferation while avoiding ferroptotic death through multilayered protective mechanisms involving glutathione systems, alternative antioxidants, and microenvironmental support.

The most striking finding is the prevalence of paradoxes that challenge conventional understanding. TNBC exhibits high ferroptosis sensitivity yet survives through microenvironmental protection from CAFs and adipocytes. The ACSL4 and UBIAD1/CoQ10 paradoxes suggest that cellular redox homeostasis operates within narrow boundaries that we don’t fully comprehend. These contradictions likely explain why ferroptosis-inducing monotherapies have failed to advance clinically despite compelling preclinical data.

Subtype-specific vulnerabilities offer the clearest path forward. The LAR TNBC subtype’s extreme sensitivity, luminal cancers’ dependence on xCT during CDK4/6 inhibitor treatment, and HER2+ tumors’ vulnerability in resistant states provide specific therapeutic windows. However, exploitation requires overcoming the redundancy problem—cancer cells employ at least six parallel protection systems (GPX4, FSP1, transsulfuration, lysosomal storage, microenvironmental supply, and MUFA incorporation), making single-target approaches futile.

*Clinical Translation Barriers*: Current strategies to exploit ferroptosis are summarized in Table 4. However, three fundamental obstacles prevent clinical advancement: (1) pharmacological—GPX4 inhibitors show poor bioavailability and hepatotoxicity; (2) biological—the tumor microenvironment provides metabolic sanctuary that cell-autonomous targeting cannot overcome; (3) selectivity—we cannot distinguish cancer from normal cell ferroptosis requirements. The promising synergies with immunotherapy and PDT face their own limitations, including immune cell toxicity and tissue penetration constraints.

*Future Priorities*: Rather than pursuing additional mechanistic studies, the field requires: (1) biomarker development to identify ferroptosis-primed tumors; (2) combination strategies that simultaneously target multiple resistance mechanisms while protecting normal tissues; (3) clinical trials in specific contexts where ferroptosis vulnerability is maximized (resistant disease, specific subtypes); (4) realistic assessment of which findings can translate versus those limited by fundamental biological constraints.

The metabolic regulation of ferroptosis in breast cancer ultimately demonstrates that targeting cell death pathways requires understanding not just the death mechanism itself, but the entire metabolic ecosystem that determines cellular fate. Success will come not from stronger ferroptosis inducers, but from strategic exploitation of context-specific vulnerabilities while managing the inevitable resistance mechanisms that evolution has provided cancer cells.

## Figures and Tables

**Table 1:** Summary of Glutathione and Cysteine Metabolism in Breast Cancer Ferroptosis Regulation.

Pathway/Mechanism	Clinical Status	Critical Gaps/Paradoxes	Key References
GPX4-GSH Axis	RSL3: preclinical only; hepatotoxicity at therapeutic doses	TNBC shows both xCT/GPX4 addiction AND high ferroptosis sensitivity; mechanism unclear	[11,17,18,19,20,21,42]
Cystine Import	Erastin: preclinical; poor bioavailability	TAM-supplied cysteine may override cell-autonomous targeting	[22,23,24,25,26,43,44,45]
Transsulfuration	No CBS inhibitors in development	Both import and synthesis can be simultaneously upregulated	[27,28,29,30,46]
Lysosomal Storage	CysRx: proof-of-concept only	Enhancing lysosomal cystine paradoxically increases ferroptosis	[31,32]
Alternative GPX4-Independent	No FSP1 inhibitors in trials	Relative contribution vs. GPX4 pathway undefined in breast cancer	[33,34,35,36,37]
Nucleotide Competition	No selective RNR modulators	Cannot target without affecting normal cell proliferation	[38,39,40,41]

**Table 2:** TNBC is heterogeneous, with subtypes showing varying ferroptosis responses [96].

TNBC Subtype	Ferroptosis Sensitivity	Key Regulatory Features
Luminal Androgen Receptor (LAR)	High	Upregulated GPX4, OxPE, glutathione metabolism
Mesenchymal (MES)	Moderate	Enriched iron metabolism, low FA/ROS activity
Immunomodulatory (IM) and Basal-Like Immune-Suppressed (BLIS)	Low	Minimal ferroptosis features, other cell death pathways dominant

**Table 3:** Oncogene and Tumor Suppressor Regulation of Ferroptosis in Breast Cancer.

Gene/ Pathway	Category	Effect on Ferroptosis	Primary Mechanisms	Clinical Relevance	Key References
RAS	Oncogene	Strong Resistance	ETS1→xCT upregulation NRF2 activation; FASN-HIF1α→MUFA FSP1 induction	Common in aggressive BC	[103,104,105,106,107,108,109,110,125,126]
mTORC1	Oncogene	Context-dependent	SREBP1-SCD1→MUFA Suppresses ferritinophagy p62-KEAP1-NRF2 Glutaminolysis paradox	Activated in most BC	[111,112,113,114,115,116,127,128,129,130]
p53	Tumor Suppressor	Promotes	Direct xCT repression SAT1→ALOX15 GLS2→glutamine depletion Acetylation-dependent	Lost in 30% BC	[39,117,118,119,131,132,133,134,135,136]
BRCA1	Tumor Suppressor	Differential	Erastin resistance GPX4 inhibitor sensitive VDAC3-dependent	5–10% hereditary BC	[121]
PTEN	Tumor Suppressor	Loss→Resistance	AKT→GSK3β→NRF2 xCT upregulation Pan-cancer mechanism	Lost in 30–40% BC	[122,123,137]
RB	Tumor Suppressor	Loss→Sensitivity	E2F→ACSL4 upregulation Increased PUFA incorporation	Lost in TNBC	[124]
HER2	Oncogene	Context-dependent	Baseline resistance Sensitivity when inhibited	Amplified 15–20% BC	[101,102]
ER	Nuclear Receptor	Resistance	MBOAT1 upregulation ELOVL2→AdA synthesis	Positive in 70% BC	[48,66,98]

**Table 4:** Ferroptosis-Targeting Therapeutic Strategies in Breast Cancer.

Strategy	Target/ Mechanism	Representative Agents	Development Status	Efficacy	Major Limitations	Key References
GPX4 Direct Inhibition	GPX4 enzymatic activity	RSL3 ML162 FIN56	Preclinical only	60–90% growth inhibition in vitro	Poor bioavailability (t½ < 2 h), hepatotoxicity	[19,42,95,101,174]
Cystine Import Blockade	System xc^−^ (SLC7A11)	Erastin IKE Sulfasalazine	Preclinical; Sulfasalazine FDA-approved	Variable (30–70% inhibition)	Poor solubility, compensatory CBS upregulation	[22,24,26,28,43]
Iron Overload/Ferritinophagy	NCOA4 FTH1 iron import	Sorafenib-NPs CT-1 Salinomycin	Preclinical	70–80% tumor reduction	Systemic iron toxicity	[15,16,175,176]
ACSL4 Modulation	PUFA incorporation	No specific inhibitors	Target identified	Context-dependent	Dual role, no selective agents	[52,54,56,67,177,178]
SCD1 Inhibition	MUFA synthesis blockade	A939572 CAY10566	Preclinical	Sensitizes to ferroptosis	Normal tissue toxicity	[63,64,137]
Selenium Manipulation	GPX4 cofactor	Sodium selenite	Preclinical	TNBC-specific toxicity	Narrow therapeutic window	[147,148,179,180,181]
FSP1 Inhibition	CoQ10-ubiquinol system	iFSP1	Early preclinical	Limited single-agent	Redundancy with GPX4	[35,36,149,182,183,184]
Immunotherapy Combination	PD-1/PD-L1 + ferroptosis	RSL3/Erastin + ICIs	Preclinical	60–90% tumor inhibition	T-cell toxicity	[160,161,162,165]
PRMT5 Inhibition	KEAP1 methylation	GSK3326595	Phase I (not BC)	60–80% with anti-PD-1	Not ferroptosis-specific	[162,185]
PDT Combination	ROS + ferroptosis	Ce6/PpIX + light	Preclinical	>95% local control	Limited penetration	[166,167,168,171,186,187]
Lipid/Metabolic Intervention	Lipid availability, glucose	Dietary GLUT inhibitors	Observational/Preclinical	Context-dependent	Patient compliance	[69,141,145,188]
Nanoparticle Delivery	Targeted delivery	Fe^3+^-NPs glutathione-scavenging	Preclinical	90% tumor inhibition	Manufacturing complexity	[45,149,165,189]
Hormone Therapy Combination	ER/HER2 + ferroptosis	Tamoxifen/Lapatinib + GPX4i	Proof of concept	Enhanced in resistant	Requires resistance	[98,99,101,102,190]
UBIAD1/CoQ10 Exploitation	Antioxidant paradox	No specific agents	Concept only	Unknown	Mechanism unclear	[150,151]

## Data Availability

No new data were created or analyzed in this study. Data sharing is not applicable to this article.

## Abbreviations

The following abbreviations are used in this manuscript:

AA	Arachidonic Acid
ACSL3	Acyl-CoA Synthetase Long-Chain Family Member 3
ACSL4	Acyl-CoA Synthetase Long-Chain Family Member 4
AdA	Adrenic Acid
AGPS	Alkylglycerone Phosphate Synthase
ALOX15	Arachidonate 15-Lipoxygenase
ATGL	Adipose Triglyceride Lipase
BH4	Tetrahydrobiopterin
CAF	Cancer-Associated Fibroblast
CBS	Cystathionine Beta-Synthase
CISD1	CDGSH Iron Sulfur Domain 1
CTH	Cystathionine Gamma-Lyase
CTNS	Cystinosin
DGLA	Dihomo-Gamma-Linolenic Acid
DLD	Dihydrolipoamide Dehydrogenase
DMT1	Divalent Metal Transporter 1
ER	Estrogen Receptor
FADS1	Fatty Acid Desaturase 1
FADS2	Fatty Acid Desaturase 2
FASN	Fatty Acid Synthase
FERscore	Ferroptosis Score
FSP1	Ferroptosis Suppressor Protein 1
FTH1	Ferritin Heavy Chain 1
G6PD	Glucose-6-Phosphate Dehydrogenase
GCH1	GTP Cyclohydrolase 1
GGT1	Gamma-Glutamyltransferase 1
GLA	Gamma-Linolenic Acid
GLS2	Glutaminase 2
GPX4	Glutathione Peroxidase 4
GSH	Glutathione
HERC2	HECT Domain and RCC1-Like Domain-Containing Protein 2
HIF1α	Hypoxia-Inducible Factor 1-Alpha
HK2	Hexokinase 2
HSL	Hormone-Sensitive Lipase
ICD	Immunogenic Cell Death
IFNγ	Interferon-Gamma
IKE	Imidazole Ketone Erastin
IL-6	Interleukin-6
KGDH	Alpha-Ketoglutarate Dehydrogenase
LA	Linoleic Acid
LAR	Luminal Androgen Receptor
LIP	Labile Iron Pool
LPCAT	Lysophosphatidylcholine Acyltransferase
MBOAT	Membrane-Bound O-Acyltransferase
MGL	Monoglyceride Lipase
MFSD12	Major Facilitator Superfamily Domain-Containing Protein 12
MUFA	Monounsaturated Fatty Acid
NADPH	Nicotinamide Adenine Dinucleotide Phosphate
NCOA4	Nuclear Receptor Coactivator 4
NRF2	Nuclear Factor Erythroid 2-Related Factor 2
OxPE	Oxidized Phosphatidylethanolamine
PC	Phosphatidylcholine
PDK4	Pyruvate Dehydrogenase Kinase 4
PD-L1	Programmed Death-Ligand 1
PE	Phosphatidylethanolamine
PKA	Protein Kinase A
PKM2	Pyruvate Kinase M2
PLA2	Phospholipase A2
PPP	Pentose Phosphate Pathway
PRMT5	Protein Arginine Methyltransferase 5
PUFA	Polyunsaturated Fatty Acid
RNR	Ribonucleotide Reductase
ROS	Reactive Oxygen Species
SAT1	Spermidine/Spermine N1-Acetyltransferase 1
SCD	Stearoyl-CoA Desaturase
SREBP1	Sterol Regulatory Element-Binding Protein 1
STEAP3	Six-Transmembrane Epithelial Antigen of Prostate 3
TAM	Tumor-Associated Macrophage
TCA	Tricarboxylic Acid
TF	Transferrin
TFR1	Transferrin Receptor 1
TNBC	Triple-Negative Breast Cancer
TOS	D-α-Tocopherol Succinate
TrxR1	Thioredoxin Reductase 1
UBIAD1	UbiA Prenyltransferase Domain-Containing Protein 1
VDAC3	Voltage-Dependent Anion Channel 3
xCT	Cystine/Glutamate Antiporter (SLC7A11)
